# Mitochondrial Distress Signals at the Heart–Liver Interface: Molecular Links Between MASLD and Heart Failure

**DOI:** 10.3390/ijms27177734

**Published:** 2026-08-28

**Authors:** Xing Yang, Kun Cheng, Chen Chen, Yuxin Zhang, Dao Wen Wang

**Affiliations:** 1 Division of Cardiology, Department of Internal Medicine, Tongji Hospital, Tongji Medical College, State Key Laboratory for Diagnosis and Treatment of Severe Zoonotic Infectious Diseases, Huazhong University of Science and Technology, Wuhan 430030, China; stella0yx@163.com (X.Y.);; 2 Hubei Key Laboratory of Genetics and Molecular Mechanism of Cardiologic Disorders, Huazhong University of Science and Technology, Wuhan 430030, China; 3 Hepatic Surgery Centre, Tongji Hospital, Tongji Medical College, Huazhong University of Science and Technology, Wuhan 430030, China; ck1597055624@163.com

**Keywords:** metabolic dysfunction-associated steatotic liver disease, heart failure, mitochondrial dysfunction, mitochondrial distress signaling, cardio-hepatic crosstalk

## Abstract

Metabolic dysfunction-associated steatotic liver disease (MASLD) and heart failure (HF) frequently coexist within a shared cardiometabolic environment, yet their mitochondrial abnormalities are stage- and phenotype-dependent rather than uniform. In MASLD, mitochondrial adaptation evolves from increased oxidative metabolism in early steatosis toward impaired respiratory flexibility, oxidative stress, and defective quality control with disease progression, whereas the failing myocardium develops reduced energetic reserve and altered substrate utilization. These organ-specific disturbances can modify mitochondria-linked metabolites, mitochondrial damage-associated molecular patterns, stress-responsive endocrine mediators, and extracellular vesicle-associated mitochondrial cargo. However, similar mitochondrial abnormalities or circulating signals in the liver and heart do not by themselves establish direct inter-organ communication. This review distinguishes shared systemic drivers and organ-intrinsic mitochondrial stress from source-resolved cardio-hepatic signaling, highlighting hepatic ketogenesis, fibroblast growth factor 21 (FGF21), mitochondrial DNA (mtDNA)-dependent inflammatory pathways, and extracellular vesicle-mediated cargo transfer as mechanistically distinct examples with different levels of evidence. We further discuss biomarker limitations, HF-related hemodynamic liver injury, and therapeutic strategies ranging from established cardiometabolic unloading to emerging mitochondria-centered interventions. A stage-, phenotype-, and source-resolved framework may improve interpretation of mitochondrial signals and guide future mechanistic and translational studies in the MASLD–HF overlap.

## 1. Introduction

Heart failure (HF) and metabolic dysfunction-associated steatotic liver disease (MASLD) are prevalent chronic disorders that frequently coexist in the setting of systemic cardiometabolic dysfunction [1,2]. HF affects more than 64 million individuals worldwide and remains a major cause of morbidity, mortality, and healthcare burden [3], whereas MASLD affects approximately 30–40% of adults globally [4]. In 2023, the nomenclature of nonalcoholic fatty liver disease (NAFLD) was revised to MASLD, which is defined by hepatic steatosis together with at least one cardiometabolic risk factor, thereby shifting the framework from one based largely on exclusion to one based on positive metabolic criteria [5]. Because a substantial body of literature was published before adoption of the current nomenclature, NAFLD and nonalcoholic steatohepatitis (NASH) are retained when referring to study populations or experimental models defined under the previous criteria, whereas MASLD and metabolic dysfunction-associated steatohepatitis (MASH) are used for current disease concepts.

MASLD is increasingly recognized as an important cardiovascular risk condition [1,6,7,8]. Patients with MASLD have increased risks of cardiovascular disease (CVD), including arrhythmias, cardiomyopathy, and HF [9,10,11]. A meta-analysis of more than 11 million individuals found an approximately 1.5-fold higher long-term risk of incident HF among individuals with NAFLD [10]. Conversely, HF can impair hepatic structure and function through reduced cardiac output, hepatic hypoperfusion, venous congestion, neurohumoral activation, and systemic inflammation [12]. In patients with coexisting metabolic liver disease, these hemodynamic and inflammatory stresses may further compromise hepatic metabolic reserve. Importantly, however, HF-related hepatic injury, including congestive or hypoxic injury, should not be equated with histological progression of MASLD/MASH. Thus, the coexistence of MASLD and HF reflects a clinically important overlap shaped by shared systemic risk factors as well as selected organ-to-organ and hemodynamic interactions.

Proposed mechanisms linking MASLD and HF include insulin resistance, lipotoxicity, chronic low-grade inflammation, oxidative stress, endothelial dysfunction, adipokine imbalance, renin–angiotensin–aldosterone system activation, and gut-derived inflammatory signals [2,9,12,13,14]. Many of these factors can affect the liver and heart in parallel without requiring sequential transmission from one organ to the other. Mitochondria are positioned at an important intersection of these processes because they integrate substrate metabolism, redox homeostasis, calcium handling, innate immune signaling, cell death pathways, and the generation or release of extracellular mediators.

Both the heart and liver depend on mitochondrial homeostasis to sustain normal organ function, yet their distinct physiological roles create different forms of vulnerability. The continuously contracting heart has a high adenosine triphosphate (ATP) demand and relies heavily on oxidative phosphorylation (OXPHOS) and fatty acid oxidation (FAO) [15,16]. Across HF phenotypes and cardiac injury models, mitochondrial abnormalities include impaired electron transport chain (ETC) activity, altered substrate utilization, excessive reactive oxygen species (ROS) generation, calcium dysregulation, and defective mitochondrial quality control [17,18,19]. By contrast, the liver functions as a systemic metabolic hub that coordinates FAO, lipogenesis, ketogenesis, glucose metabolism, amino acid turnover, and detoxification [20]. In MASLD, mitochondrial remodeling is stage-dependent: early steatosis may be accompanied by increased tricarboxylic acid (TCA)-cycle flux and respiratory activity, whereas persistent lipid overload and progression toward MASH are associated with loss of metabolic flexibility, mitochondrial uncoupling, oxidative stress, defective mitophagy, and inflammatory activation [21,22,23,24]. These findings indicate convergent mitochondrial stress in the two organs but do not imply a uniform mitochondrial phenotype or direct inter-organ transmission of mitochondrial dysfunction.

In this review, mitochondrial distress signaling is used as an operational term to describe stress-induced changes in mitochondria-derived or mitochondria-associated signals that can modify cellular responses locally or systemically. These signals include mitochondria-linked metabolites, mitochondrial damage-associated molecular patterns (mtDAMPs), stress-responsive endocrine mediators, and extracellular vesicle (EV)-associated mitochondrial cargo [25,26,27,28]. Their biological effects range from adaptive substrate exchange and stress compensation to context-dependent inflammatory or maladaptive signaling. This operational grouping does not imply that all signals share a common mitochondrial origin, are uniformly pathogenic, or arise predominantly from the liver; rather, their interpretation depends on cellular and tissue source, circulating form, recipient cell or receptor, concentration, disease context, and the strength of causal evidence. Importantly, the presence of similar mitochondrial abnormalities or circulating mediators in both organs does not by itself establish direct cardio-hepatic communication. Accordingly, this review distinguishes organ-intrinsic mitochondrial remodeling, shared systemic drivers, and source-resolved inter-organ signaling, and examines how bioenergetic adaptation, mitochondrial dynamics, quality control, and redox balance influence these processes and their translational relevance to the MASLD–HF overlap.

## 2. Literature Search Strategy

For this narrative review, a structured literature search was performed using PubMed and the Web of Science Core Collection from database inception through August 2026. Search terms were combined around four major themes: (i) MASLD/MASH and the historical terms NAFLD/NASH; (ii) HF, including heart failure with preserved ejection fraction (HFpEF) and heart failure with reduced ejection fraction (HFrEF); (iii) mitochondrial metabolism and homeostasis, including bioenergetics, mitochondrial dynamics, mitophagy, redox signaling, mitochondrial stress responses, the integrated stress response (ISR), and the mitochondrial unfolded protein response (UPRmt); and (iv) candidate circulating mediators associated with mitochondrial stress or metabolism, including mitochondrial DNA (mtDNA), mtDAMPs, EVs, succinate, acylcarnitines, ketone bodies, fibroblast growth factor 21 (FGF21), and growth differentiation factor 15 (GDF15). Additional relevant studies were identified from the reference lists of key primary articles and recent reviews.

The review focused primarily on peer-reviewed, English-language articles. Human clinical and population-based studies were prioritized when evaluating disease associations, HF phenotypes, circulating biomarkers, and therapeutic translation. Experimental animal studies were used primarily to assess organ-level and mechanistic relationships, whereas cell-based studies were included to support specific molecular mechanisms where appropriate. Particular emphasis was placed on original studies incorporating genetic, pharmacological, or other causal perturbations, and source-resolved studies were given greater weight when evaluating proposed inter-organ signaling pathways. Recent high-quality reviews were used to provide broader context and to identify relevant primary studies, while seminal earlier studies were retained where necessary. Because this article was designed as a narrative review rather than a systematic review, formal risk-of-bias assessment and quantitative evidence synthesis were not performed.

## 3. Stage- and Phenotype-Specific Mitochondrial Remodeling in MASLD and HF

Mitochondria support distinct physiological demands in the liver and heart, and the consequences of mitochondrial stress therefore differ between these organs. Hepatic mitochondria coordinate FAO, TCA cycle flux, ketogenesis, gluconeogenesis, amino acid metabolism, and redox balance, whereas cardiac mitochondria are required to sustain continuous ATP production for excitation–contraction coupling and mechanical work [23,29,30,31]. Thus, the heart is primarily constrained by its high mitochondrial energy demand, whereas the liver is primarily challenged by the need to process and redistribute metabolic substrates. Importantly, neither MASLD nor HF is characterized by a single mitochondrial phenotype. The clinical overlap between the two disorders is particularly relevant to metabolically burdened HF phenotypes, especially obesity-related HFpEF. In a recent cohort of 26,676 individuals with steatotic liver disease, MASLD was associated with a higher risk of incident HF, and 76% of newly diagnosed HF cases were HF with preserved ejection fraction (HFpEF); MASLD was specifically associated with increased HFpEF risk. Earlier prospective data also showed a high prevalence of NAFLD and advanced fibrosis among patients with HFpEF, particularly in those with obesity and diabetes [6,32]. These observations support a phenotype-resolved approach when considering mitochondrial remodeling in the cardio-hepatic setting.

In MASLD, mitochondrial remodeling follows a stage-dependent trajectory rather than a pattern of uniform bioenergetic failure. Human tracer studies demonstrated that hepatic TCA-cycle flux is markedly increased in individuals with fatty liver, consistent with an early compensatory increase in oxidative metabolism in response to substrate excess [23]. This concept was strengthened by high-resolution respirometry of human liver biopsies, which showed that obese insulin-resistant individuals with or without hepatic steatosis exhibited substantially greater maximal mitochondrial respiration than lean controls despite similar mitochondrial content. By contrast, patients with steatohepatitis showed lower maximal respiratory capacity despite increased mitochondrial mass, together with greater uncoupling, proton leak, oxidative stress, and impaired antioxidant defense [24]. Earlier biopsy-based studies similarly identified defects in respiratory chain activity in NASH [33]. Thus, human studies indicate increased oxidative metabolism in simple steatosis, whereas steatohepatitis is associated with impaired respiratory efficiency, greater proton leak, and increased oxidative stress.

This stage-dependent interpretation is particularly important when discussing advanced MASLD. Fibrosis and cirrhosis should not simply be regarded as progressively more severe versions of the mitochondrial phenotype observed in early steatosis. At these stages, mitochondrial dysfunction occurs within a broader context of persistent hepatocellular injury, inflammatory activation, fibrogenesis, altered hepatic architecture, and impaired metabolic reserve. Clinical studies comparing fibrosis stages have reported reduced mitochondrial respiratory reserve capacity and broader metabolite disturbances in patients with advanced versus mild fibrosis, although measurements in such studies are not always hepatocyte-specific [34]. Thus, the most firmly established human mitochondrial transition remains the shift from enhanced oxidative adaptation in simple steatosis toward loss of respiratory flexibility and oxidative injury in steatohepatitis, with advanced fibrosis representing a more complex state of metabolic and structural decompensation.

Cardiac mitochondrial remodeling is likewise phenotype-dependent. In dilated cardiomyopathy (DCM) and advanced HFrEF, impaired high-energy phosphate metabolism is well established. Myocardial phosphocreatine-to-ATP ratios are reduced in ischemic and idiopathic dilated cardiomyopathy, and a lower ratio predicts mortality in patients with dilated cardiomyopathy [35,36]. Absolute measurements further showed reductions in both phosphocreatine and ATP in severe DCM [37]. Advanced human HF is also accompanied by substantial substrate remodeling; explanted failing hearts exhibit depletion of several lipid intermediates and increased myocardial ketone utilization, consistent with greater reliance on ketone oxidation as an alternative fuel pathway in severe systolic HF [38]. Together, these observations define a characteristic pattern of energetic impairment and substrate remodeling in advanced systolic HF and underscore the need to distinguish it from other HF phenotypes.

The metabolic phenotype of HFpEF differs in important respects from that of advanced systolic HF. In a widely used two-hit experimental HFpEF model combining obesity and hypertension, cardiac ATP production becomes more dependent on FAO as insulin-stimulated glucose oxidation declines. Altered regulation of pyruvate dehydrogenase (PDH) has also been reported, including reduced PDH phosphorylation in both experimental HFpEF and myocardial samples from patients with HFpEF [39]. A complementary study showed that experimental HFpEF hearts retained high dependence on FAO but developed reduced phosphocreatine content, impaired mitochondrial respiration, increased oxidative stress, and blunted mitophagic responses compared with hearts exposed to obesity alone [40]. These findings suggest that metabolic HFpEF may involve maladaptation to chronic metabolic stress rather than simple energetic deficiency, with persistent substrate availability becoming increasingly mismatched to mitochondrial flexibility and quality control capacity. This distinction is relevant to MASLD because obesity, insulin resistance, and excess lipid delivery can impose simultaneous metabolic pressure on the liver and myocardium without implying that the mitochondrial phenotype is identical in both organs.

**Figure 1:**
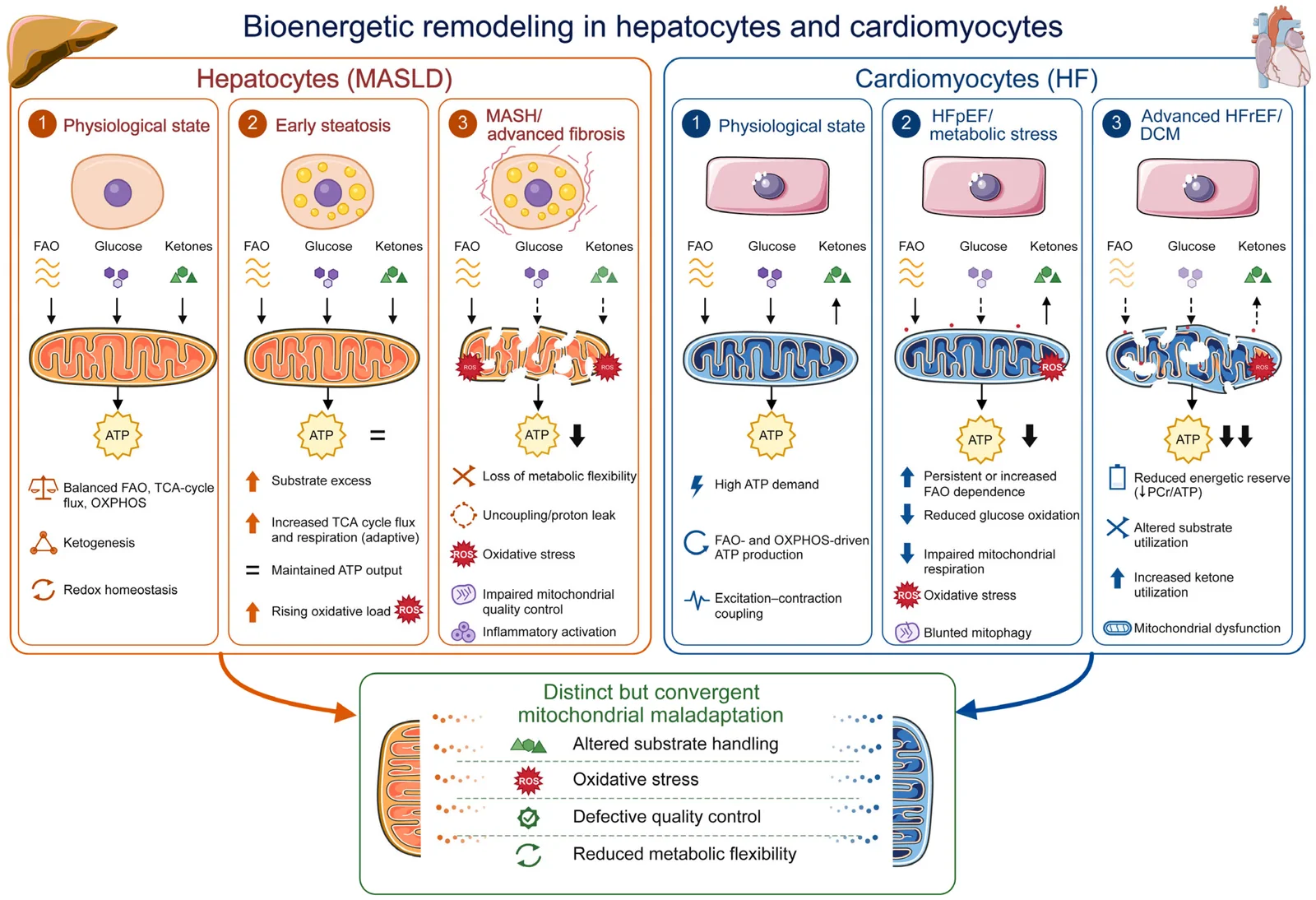
Stage- and phenotype-dependent bioenergetic remodeling in MASLD and heart failure. Early MASLD may show compensatory increases in mitochondrial substrate oxidation and respiration, whereas advanced MASH/fibrosis is associated with oxidative stress, impaired quality control, and reduced metabolic flexibility. In heart failure, mitochondrial remodeling varies across phenotypes, from metabolic stress in HFpEF to more pronounced energetic failure in advanced HFrEF/DCM. Despite these differences, both diseases converge on altered substrate utilization, oxidative stress, defective mitochondrial quality control, and reduced metabolic flexibility. The depicted stages represent conceptual patterns rather than an obligatory linear progression. For the metabolic pathways depicted above each mitochondrion, solid black arrows indicate relatively maintained or increased activity, whereas dashed black arrows indicate reduced activity. The cor-responding pathway symbols are shown in darker and lighter shades, respectively. For ketone metabolism, upward arrows in the hepatocyte panels denote ketone body production and export, whereas downward arrows in the cardiomyocyte panels denote ketone uptake and oxidation. These visual cues represent qualitative rather than quantitative changes. Abbreviations: ATP, adenosine triphosphate; DCM, dilated cardiomyopathy; FAO, fatty acid oxidation; HF, heart failure; HFpEF, heart failure with preserved ejection fraction; HFrEF, heart failure with reduced ejection fraction; MASLD, metabolic dysfunction-associated steatotic liver disease; MASH, metabolic dysfunction-associated steatohepatitis; OXPHOS, oxidative phosphorylation; PCr/ATP, phosphocreatine-to-ATP ratio; ROS, reactive oxygen species; TCA, tricarboxylic acid.

HF-associated liver injury requires a separate clinical interpretation. Reduced cardiac output, hepatic hypoperfusion, elevated right-sided filling pressures, and venous congestion can alter hepatic metabolism and produce biochemical and structural liver abnormalities. In acute decompensated HF, abnormal liver function tests are common and are associated with distinct hemodynamic profiles [41]. In patients with ischemic hepatitis, severe underlying cardiac disease and right-sided HF with passive hepatic congestion were highly prevalent, suggesting that venous congestion may increase hepatic susceptibility to superimposed hypoperfusion [42]. Chronic congestive hepatopathy is likewise observed in advanced HF and is related to recurrent congestion and impaired arterial perfusion [43]. These processes are mechanistically distinct from the metabolically initiated progression of MASLD to MASH and fibrosis. Accordingly, hepatic abnormalities in HF should be described as congestive hepatopathy, hypoxic/ischemic liver injury, or HF-associated hepatic dysfunction when supported by the clinical context, rather than being automatically interpreted as progression of MASLD. Taken together, reduced energetic reserve in the stressed myocardium and excessive substrate-processing demand in the steatotic liver represent distinct but convergent forms of mitochondrial maladaptation, whereas HF-related hepatic injury constitutes a mechanistically separate hemodynamic and hypoperfusion-related process. These stage- and phenotype-dependent patterns of mitochondrial remodeling in MASLD and HF are summarized in Figure 1.

## 4. Mitochondrial Remodeling, Quality Control, and Stress Adaptation

Mitochondrial structure, selective clearance, renewal, stress-response signaling, and redox balance form an interconnected quality-control system [44]. Fission and fusion redistribute mitochondrial contents and segregate damaged segments; mitophagy and lysosomal degradation remove damaged material; mitochondrial biogenesis restores organelle capacity; stress-response pathways adjust protein synthesis and metabolism; and antioxidant systems limit excessive oxidative injury [45]. These processes are functionally interconnected, and impairment of one component can compromise the efficiency of the others.

### 4.1. Mitochondrial Dynamics and Structural Adaptation

Mitochondrial dynamics provides a structural basis for mitochondrial adaptation to cellular stress. Dynamin-related protein 1 (DRP1) mediates mitochondrial fission, whereas mitofusin 1 and 2 (MFN1 and MFN2) mediate outer mitochondrial membrane fusion, and optic atrophy 1 (OPA1) mediates inner mitochondrial membrane fusion [46,47,48]. Basal fission supports mitochondrial segregation and quality control, while fusion maintains content exchange and respiratory competence [49]. These processes are therefore not intrinsically beneficial or harmful; their effects depend on whether structural remodeling remains coordinated with metabolic demand and organelle clearance.

Mechanical load, calcium dysregulation, ischemia, and neurohumoral activation alter mitochondrial structure in the myocardium. Combined deletion of *Mfn1* and *Mfn2* in cardiomyocytes causes mitochondrial fragmentation, respiratory dysfunction, ventricular dilation, and lethal HF [50]. Conversely, DRP1-dependent mitophagy is required for adaptation to pressure overload, indicating that fission is protective when coupled to effective mitophagy [51]. These studies show that both loss of fusion and impaired fission-dependent quality control can be detrimental to the stressed myocardium.

Lipid excess, endoplasmic reticulum (ER) stress, and oxidative stress similarly remodel mitochondrial structure in hepatocytes. Reducing excessive fission can attenuate steatosis and oxidative injury in experimental fatty liver [52], yet complete or sustained disruption of mitochondrial division can also be maladaptive. Hepatocyte-specific deletion of mitochondrial fission factor (MFF) aggravates high-fat diet-induced NASH, while liver-targeted *Drp1* knockdown fails to reverse established NASH and enhances the mitochondrial integrated stress response (mitoISR) [53,54]. Hepatic MFN2 deficiency disrupts ER-mitochondria phospholipid transfer, induces ER stress, and promotes a NASH-like phenotype [55], whereas exercise-induced MFN2 enhances lipid droplet-mitochondria communication and FAO [56].

Mitochondrial dynamics is thus better understood as structural plasticity than as a binary fission-fusion switch. Excessive fragmentation is associated with respiratory inefficiency and ROS generation, whereas insufficient fission can prevent damaged segments from being segregated and removed [51,57,58,59]. OPA1-dependent cristae remodeling influences respiratory efficiency and apoptotic susceptibility [60,61], and ER-mitochondria contact sites coordinate constriction, calcium transfer, and lipid exchange; ER tubules can mark mitochondrial division sites before DRP1 recruitment [62]. In hepatocytes, these structural processes also intersect with lipid-droplet communication and the mitochondrial integrated stress response [56,63].

### 4.2. Mitophagy and Mitochondrial Renewal

Mitophagy removes damaged or superfluous mitochondria before they persistently generate ROS, release mtDNA, and activate inflammatory signaling and cell death pathways [64]. In the canonical PTEN-induced kinase 1 (PINK1)/Parkin pathway, mitochondrial depolarization stabilizes PINK1 on the outer mitochondrial membrane; PINK1 then recruits and activates Parkin, thereby promoting the ubiquitination of mitochondrial surface proteins [65,66,67]. Autophagy receptors, including optineurin (OPTN), nuclear dot protein 52 (NDP52), and sequestosome 1 (SQSTM1/p62), then connect damaged mitochondria to microtubule-associated protein 1 light chain 3 (LC3)-positive autophagic membranes [68]. Receptor-mediated pathways involving FUN14 domain-containing protein 1 (FUNDC1), BCL2/adenovirus E1B 19 kDa protein-interacting protein 3 (BNIP3), or BCL2/adenovirus E1B 19 kDa protein-interacting protein 3-like (NIX/BNIP3L) provide additional routes under hypoxia and nutrient stress [69,70].

Mitophagic demand rises as mitochondrial damage accumulates. Fission facilitates segregation of damaged components, whereas incomplete mitophagic flux causes their persistence [51,59,71,72]. In the heart, PINK1 deficiency produces mitochondrial dysfunction, oxidative stress, pathological cardiac hypertrophy, and left ventricular dysfunction [73]. Parkin deficiency worsens post-infarction injury, whereas Parkin overexpression protects cardiomyocytes from hypoxia-induced death [74]. Cytosolic p53 also suppresses Parkin-mediated mitophagy and promotes mitochondrial dysfunction [75]. These findings support mitochondrial surveillance as an important determinant of cardiac stress tolerance.

In nutrient-stressed hepatocytes, chronic lipid overload promotes depolarization, oxidative injury, and mtDNA damage. Mitophagy-reporter studies show that mitochondrial clearance declines early during NAFLD development and that liver-specific Parkin deletion accelerates steatosis, inflammation, and fibrosis [76]. In NASH, a tumor necrosis factor-α (TNF-α)/MYC-interacting zinc-finger protein 1 (MIZ1) feedback loop suppresses hepatocyte mitophagy, increases the accumulation of dysfunctional mitochondria, and sustains macrophage-derived inflammatory signaling [77]. Impaired mitophagy can therefore participate in the transition from metabolic overload to steatohepatitis rather than merely reflecting late-stage injury.

Importantly, mitochondrial fragmentation or changes in the steady-state abundance of PINK1, Parkin, or LC3 do not by themselves establish altered mitophagic flux. Flux-oriented approaches that monitor mitochondrial delivery to lysosomes over time, including mito-QC and mitochondrial-targeted Keima (mt-Keima) reporters, provide a more direct assessment of mitochondrial turnover [78,79].

Mitochondrial quality control also requires renewal. Peroxisome proliferator-activated receptor-γ coactivator 1α (PGC-1α)-driven biogenesis replenishes the mitochondrial pool after organelle removal [80], while mitochondria-derived vesicles can selectively transport mitochondrial cargo to lysosomes before whole-organelle mitophagy is engaged [81]. PINK1 and Parkin also participate in this vesicular pathway under oxidative stress [82]. When segregation, lysosomal degradation, and biogenesis are no longer matched, dysfunctional mitochondria accumulate and become sustained sources of metabolic, oxidative, and inflammatory signals.

### 4.3. Mitochondrial Stress Responses

Mitochondrial perturbation does not necessarily lead immediately to organelle elimination. Cells first activate coordinated stress-response programs that adjust protein synthesis, metabolism, proteostasis, and mitochondrial turnover according to the type and intensity of the insult. Mitochondrial protein import efficiency is an important component of this surveillance system. During moderate proteotoxic or metabolic stress, impaired mitochondrial import can redirect activating transcription factor 5 (ATF5) toward the nucleus and activate transcriptional programs associated with the UPRmt. ATF5-dependent UPRmt signaling contributes to the maintenance and recovery of mitochondrial function under stress [83,84].

In parallel, disturbances in mitochondrial membrane potential, protein import, ROS, or iron availability can activate the OMA1 zinc metallopeptidase (OMA1)–DAP3-binding cell death enhancer 1 (DELE1)–heme-regulated eIF2α kinase (HRI) pathway. Cytosolic DELE1 activates HRI, leading to phosphorylation of eukaryotic translation initiation factor 2 alpha (eIF2α), attenuation of global protein synthesis, and induction of activating transcription factor 4 (ATF4)-dependent transcription as part of the ISR [85,86].

The mitoISR is closely connected to mitochondrial quality control. A recent mechanistic study showed that under relatively mild mitochondrial stress, DELE1–HRI-dependent translational attenuation preserves mitochondrial protein import efficiency and limits premature PINK1-dependent mitophagy. Under severe depolarizing stress, mitochondrial import becomes markedly impaired despite ISR activation, allowing PINK1 accumulation and robust mitophagy [87]. A genetic model of mitochondrial cardiomyopathy further showed that OMA1–DELE1-dependent ISR activation limits ferroptotic injury and cardiac dysfunction [88]. These findings indicate that mitochondrial stress responses are dynamically coordinated with organelle repair and removal rather than operating as independent pathways.

Mitochondrial stress responses can also generate extracellular signals that extend adaptation beyond the stressed cell. FGF21 and GDF15 are among the best-characterized stress-responsive endocrine mediators associated with mitochondrial stress. In experimental mitochondrial myopathy, activation of a mechanistic target of rapamycin complex 1 (mTORC1)-regulated mitoISR is accompanied by induction of FGF21 and GDF15 together with extensive remodeling of one-carbon and amino-acid metabolism [89]. These findings support an endocrine component of the mitochondrial stress response rather than indicating that FGF21 and GDF15 are specific markers of mitochondrial injury.

The biological effects of these mitokines are nevertheless tissue- and context-dependent. In models of mild-to-moderate cardiac oxidative phosphorylation dysfunction, endogenous FGF21 regulates part of the mitoISR and supports metabolic adaptation, whereas its contribution becomes less evident under severe mitochondrial dysfunction [90]. More recently, Mia et al. combined tissue-specific genetic models with antisense oligonucleotide treatment and showed that liver-derived FGF21 contributes to cardiac FGF21 signaling during pressure overload [91]. Thus, endogenous FGF21 can participate in both adaptive mitochondrial stress responses and context-dependent inter-organ signaling. GDF15 appears to have a broader role as a systemic stress signal. Muscle-specific mitochondrial dysfunction induces GDF15 release and GDF15-dependent changes in appetite and systemic metabolic adaptation [92], while activation of the ISR in adipocytes induces GDF15 through ATF4 and C/EBP homologous protein (CHOP) [93]. Because GDF15 can be produced by multiple stressed tissues [94], its role in MASLD and HF is currently better considered as a stress-responsive systemic signal than as an established liver-to-heart mediator. The circulating behavior and potential cardio-hepatic relevance of FGF21 and GDF15 are considered further in the section on mitochondria-associated systemic signals. Neither FGF21 nor GDF15 is specific to mitochondrial injury, because both can also be induced by broader metabolic and cellular stress programs.

### 4.4. Redox Homeostasis and Oxidative Stress

Bioenergetic-redox coupling determines whether mitochondrial metabolism supports adaptive signaling or oxidative injury. Under physiological conditions, substrate oxidation, electron transfer, ATP synthesis, and antioxidant buffering are closely matched. Low levels of mitochondrial ROS can serve signaling functions, but when electron flux exceeds respiratory capacity or antioxidant defense becomes insufficient, controlled redox signaling shifts to oxidative stress [95,96]. Impaired respiration then increases electron leakage, while ROS further damage respiratory proteins, membrane lipids, cardiolipin, and mtDNA [95,97,98].

In models of pressure overload, myocardial infarction, and genetic cardiomyopathy, mitochondrial oxidative stress is associated with mtDNA damage, respiratory chain dysfunction, hypertrophy, and ventricular failure [99,100,101,102]. Cardiomyocyte superoxide dismutase 2 (SOD2) deficiency further causes mitochondrial lipid peroxidation, respiratory impairment, and lethal dilated cardiomyopathy [103]. These studies support an important protective role for mitochondrial antioxidant capacity during cardiac stress.

In MASLD, respiratory-chain defects, ultrastructural abnormalities, cardiolipin oxidation, and impaired oxidative metabolism have been reported, particularly during progression to steatohepatitis [23,24,33,104]. These abnormalities are accompanied by increased oxidative and inflammatory stress. Mitochondrial ROS and ROS generated by nicotinamide adenine dinucleotide phosphate (NADPH) oxidase 4 (NOX4) can activate nuclear factor erythroid 2-related factor 2 (NRF2)-dependent antioxidant defenses that restrain obesity-associated NASH progression [105]. Accordingly, sustained ROS production that exceeds antioxidant capacity is more consistently associated with tissue injury than transient redox signaling.

In the liver, mitochondrial oxidative stress can amplify inflammatory and fibrotic signaling, whereas HF-associated hypoperfusion and venous congestion can increase hepatic hypoxia and oxidative stress. These hemodynamic effects should be distinguished from direct molecular heart–liver signaling. The 66-kDa isoform of SHC-transforming protein 1 (p66Shc), for example, increases mitochondrial ROS production and NLR family pyrin domain-containing 3 (NLRP3) inflammasome activation in experimental liver fibrosis [106]. Hepatocyte mitochondrial dysfunction can also influence Kupffer cell and hepatic stellate cell activation during steatohepatitis [107].

**Figure 2:**
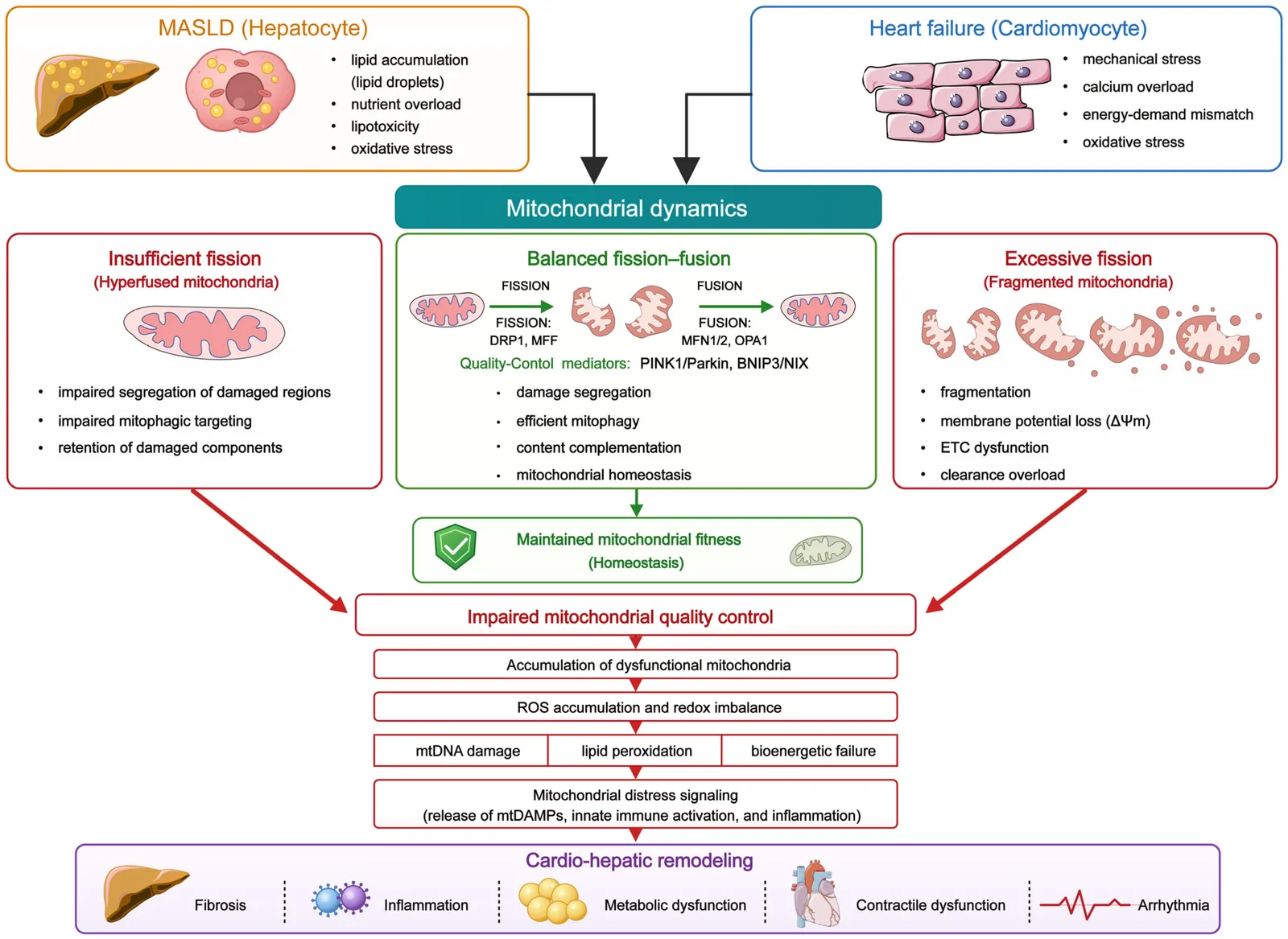
Context-dependent mitochondrial dynamics and quality-control failure in MASLD and HF. Balanced fission–fusion dynamics support mitochondrial quality control, whereas both insufficient fission and excessive fragmentation can impair the segregation and clearance of damaged mitochondria. Persistent quality-control failure promotes oxidative stress, mitochondrial damage, bioenergetic dysfunction, and mitochondrial distress signaling. Abbreviations: BNIP3, BCL2/adenovirus E1B 19 kDa protein-interacting protein 3; DRP1, dynamin-related protein 1; ETC, electron transport chain; HF, heart failure; MASLD, metabolic dysfunction-associated steatotic liver disease; MFF, mitochondrial fission factor; MFN1/2, mitofusin 1 and 2; mtDAMPs, mitochondrial damage-associated molecular patterns; mtDNA, mitochondrial DNA; NIX, BCL2/adenovirus E1B 19 kDa protein-interacting protein 3-like; OPA1, optic atrophy 1; PINK1, PTEN-induced kinase 1; ROS, reactive oxygen species; ΔΨm, mitochondrial membrane potential.

Persistent mitochondrial damage can increase the cytosolic exposure or extracellular release of mtDNA, oxidized lipids, and other mitochondria-associated signals, thereby linking organelle dysfunction to intracellular and extracellular stress signaling (Figure 2).

## 5. Mitochondria-Associated Signals in Cardio-Hepatic Communication

Mitochondrial stress becomes relevant to inter-organ communication when it alters the circulating abundance, localization, or biological activity of metabolites, mtDAMPs, EV-associated cargo, or stress-responsive endocrine factors. These mediators operate within a broader cardiometabolic environment shaped by the liver, heart, adipose tissue, skeletal muscle, immune system, kidney, and gut. Their interpretation therefore depends on tissue and cellular origin, circulating form, recipient cell or receptor, and disease stage. Importantly, similar mitochondrial abnormalities in the liver and heart do not themselves establish direct inter-organ communication, and evidence for source-resolved causality varies substantially among the four signal classes discussed below.

### 5.1. Mitochondria-Linked Metabolic Signals

Mitochondria-linked metabolites may act as adaptive fuels, systemic indicators of substrate imbalance, or receptor-active signaling molecules. In MASLD and HF, alterations in TCA-cycle flux, FAO, ketogenesis, and redox balance can influence circulating succinate, acylcarnitines, β-hydroxybutyrate, and related metabolites; however, circulating abundance alone does not establish tissue origin or distant-organ causality.

Succinate illustrates the importance of cell-type and context specificity. Succinate accumulation during hypoxia, inflammation, nutrient excess, or impaired electron transport can activate succinate receptor 1 (SUCNR1), also known as G protein-coupled receptor 91 (GPR91). SUCNR1 signaling can support adaptive responses in metabolically stressed hepatocytes, whereas activation in hepatic stellate cells promotes fibrogenic signaling [108,109]. In cardiomyocytes, exogenous succinate can induce GPR91-dependent hypertrophic signaling [110]. Because succinate is produced and consumed by multiple tissues, these findings support context-dependent signaling rather than a specifically liver-derived pathway to HF.

Acylcarnitines are better interpreted as multisource metabolic readouts. Their accumulation can reflect incomplete β-oxidation [111], and altered circulating acylcarnitine profiles have been reported in NAFLD and in both HFpEF and HFrEF [112,113,114]. However, circulating concentrations integrate metabolism by the liver, skeletal muscle, myocardium, and adipose tissue, together with renal and hepatic clearance. End-stage failing human myocardium can even show depletion of myocardial acylcarnitines despite elevations of selected circulating species [115]. Thus, without isotope tracing or organ-specific perturbation, circulating acylcarnitines mainly indicate systemic incomplete FAO and metabolic inflexibility.

β-Hydroxybutyrate represents a more clearly adaptive substrate. Circulating ketone bodies are generated predominantly through hepatic mitochondrial ketogenesis and can subsequently be taken up and oxidized by extrahepatic tissues, including the myocardium [38,116]. Advanced HFrEF is associated with increased myocardial ketone utilization [38], and acute β-hydroxybutyrate administration increases cardiac output in patients with chronic HFrEF [117]. β-Hydroxybutyrate can also suppress NLRP3 inflammasome activation in immune cells [118]. Hepatic ketogenesis and cardiac ketone utilization therefore constitute a physiologically relevant liver-to-heart substrate pathway, although increased ketone use in HF is more consistent with metabolic adaptation than with a pathogenic MASLD-derived signal.

Taken together, mitochondria-linked metabolites should be interpreted according to source specificity, recipient-cell biology, and functional context rather than circulating concentration alone.

### 5.2. Mitochondrial DAMPs

mtDAMPs are mitochondrial components that acquire signaling activity when displaced from their normal intracellular compartment or released extracellularly. They include mtDNA, oxidized mtDNA, mitochondrial transcription factor A (TFAM), cardiolipin, ATP, mitochondrial N-formyl peptides, and oxidized mitochondrial lipids [26,119]. Their effects depend strongly on localization, allowing the same mitochondrial constituent to participate in cell-autonomous, local paracrine, or potentially systemic signaling.

Among mtDAMPs, mtDNA is the best-characterized example. Mitochondrial oxidative injury and membrane permeabilization can promote mtDNA release through mechanisms involving BCL-2-associated X protein (BAX), BCL-2 antagonist/killer 1 (BAK1), and voltage-dependent anion channel (VDAC) oligomerization [120,121]. Cytosolic mtDNA activates cyclic GMP–AMP synthase (cGAS)–stimulator of interferon genes (STING) signaling, promoting type I interferon production and pro-inflammatory responses, whereas extracellular mtDNA internalized into endolysosomal compartments can activate Toll-like receptor 9 (TLR9) and amplify sterile inflammation [122,123]. Oxidized mtDNA can additionally promote NLRP3 inflammasome activation [124].

In metabolic liver disease, relatively strong cell-resolved evidence supports hepatocyte-to-myeloid-cell signaling. In experimental NASH, most circulating mtDNA was associated with hepatocyte-derived microparticles capable of activating TLR9-dependent responses [27]. Lipid-stressed hepatocyte mtDNA can also activate STING–nuclear factor-κB (NF-κB) signaling in Kupffer cells, and STING deficiency attenuates hepatic inflammation and fibrosis [125]. In human NAFLD, disease-associated STING expression is enriched in hepatic macrophage populations [126]. These findings establish local and extracellular hepatocyte–immune-cell communication, although cardiac delivery of liver-derived mtDNA has not been demonstrated.

A comparable distinction applies to the heart. Pressure overload activates cardiac cytosolic DNA sensing and cGAS–STING-associated inflammatory signaling, and genetic or pharmacological inhibition attenuates adverse remodeling [127,128]. This provides causal evidence for an organ-intrinsic cardiac pathway rather than for heart-to-liver mtDNA transfer. Other mtDAMPs, including cardiolipin, extracellular ATP, and mitochondrial N-formyl peptides, can activate NLRP3, purinergic receptors, or formyl peptide receptors on innate immune cells [26,129]. Circulating mitochondrial products can also activate neutrophils through TLR9- and formyl peptide receptor-dependent mechanisms [26].

Vascular and innate immune cells may therefore act as intermediary recipients of extracellular mtDAMPs. Mitochondrial N-formyl peptides and mtDNA activate neutrophils through formyl peptide receptor 1 (FPR1)- and TLR9-dependent mechanisms [26], while studies in other disease settings show that extracellular or mislocalized mtDNA can trigger NLRP3- or TLR9-dependent endothelial inflammation and vascular dysfunction [130,131]. Their specific contribution to a source-resolved MASLD-to-HF pathway, however, remains uncertain.

### 5.3. Stress-Responsive Endocrine Mediators

Mitochondrial dysfunction can also induce transcriptional stress programs, including the ISR and UPRmt, leading to secretion of endocrine factors such as FGF21 and GDF15 [89,132]. These molecules are therefore better regarded as mitochondrial stress-responsive endocrine mediators than as mitochondria-derived cargo, and neither is specific to the liver or heart.

FGF21 provides an important example of source-dependent endocrine signaling. The liver is a major contributor to circulating FGF21 under metabolic stress, although adipose tissue and cardiomyocytes can also produce FGF21 [133]. Its signaling requires β-Klotho-containing receptor complexes, making recipient-cell responsiveness receptor-dependent [134]. Liver- and adipose-derived FGF21 can exert endocrine effects on ischemic myocardium [135], whereas cardiomyocyte-derived FGF21 can act locally during cardiac stress [136].

Source-resolved studies further support inter-organ FGF21 signaling. After myocardial infarction, an interleukin-6 (IL-6)/signal transducer and activator of transcription 3 (STAT3)/mineralocorticoid receptor/FGF21 pathway stimulates hepatic FGF21 production and contributes to cardiac repair [137]. More recently, Mia et al. combined tissue-specific genetic models with antisense oligonucleotide treatment and showed that liver-derived FGF21 contributes to cardiomyocyte FGF21 signaling during pressure overload [91]. These studies provide source-resolved evidence for hepato-cardiac FGF21 signaling in selected cardiac injury models, although its relevance to MASLD-associated HF remains uncertain.

GDF15 is mechanistically distinct. Hepatic GDF15 can be induced through CHOP-associated stress responses and can reduce food intake and improve systemic insulin sensitivity in obese mice [138,139]. Skeletal-muscle mitochondrial stress can also induce GDF15, demonstrating that circulating GDF15 is not liver-specific [140]. Recent high-sensitivity mapping indicates that GDNF family receptor alpha-like (GFRAL) expression is overwhelmingly restricted to the area postrema and nucleus of the solitary tract, with no robust expression in peripheral tissues such as the heart or liver [141]. Together with its requirement for the co-receptor rearranged during transfection (RET), this distribution supports a predominantly central mechanism for the established metabolic effects of GDF15 [142]. Thus, GDF15 is an important systemic stress-responsive mediator but currently has limited support as a direct hepatocyte-to-cardiomyocyte signal.

FGF21 and GDF15 therefore illustrate how mitochondrial dysfunction can be translated into systemic endocrine responses without direct release of mitochondrial components.

### 5.4. Extracellular Vesicles as Candidate Carriers of Mitochondrial Cargo

EVs can transport proteins, lipids, nucleic acids, metabolites, and organelle-derived components [143]. Under specific stress conditions, EV preparations have also been reported to contain mtDNA, mitochondrial RNA, mitochondrial proteins, lipids, and occasionally larger mitochondrial structures [144]. General EV-mediated communication should nevertheless be distinguished from mitochondrial-cargo-specific signaling.

In MASLD models, lipotoxic hepatocytes release EVs that act on both local and extrahepatic recipient cells. Hepatocyte-derived EVs can activate hepatic stellate cells through microRNA-mediated suppression of peroxisome proliferator-activated receptor γ (PPARγ), recruit macrophages through ceramide/sphingosine-1-phosphate-dependent mechanisms, and induce endothelial inflammation through microRNA-1-dependent signaling [145,146,147,148]. These studies establish hepatocytes as EV-producing cells and hepatic stellate cells, macrophages, and endothelial cells as recipient populations, but the experimentally defined cargo is predominantly non-mitochondrial.

More directly relevant to mitochondrial signaling, circulating microparticles from hepatocytes in experimental NASH can contain mtDNA and other mitochondrial material. Most circulating mtDNA in this model was associated with hepatocyte-derived particles, and particle depletion markedly reduced the TLR9-activating capacity of plasma [27]. This provides source-resolved evidence linking hepatocyte mitochondrial injury to extracellular particle-mediated immune signaling, although the demonstrated recipient cells were predominantly myeloid.

Direct evidence that hepatocyte-derived EVs carrying mitochondrial cargo are taken up by defined cardiac cell populations and causally modify an HF phenotype remains scarce. Evidence in the reverse direction is similarly limited. EV-mediated mitochondrial cargo transfer should therefore be considered a candidate transport mechanism rather than an established bidirectional MASLD–HF pathway.

Methodological limitations are particularly important in this field. EV preparations may contain lipoproteins, protein aggregates, cell fragments, and other non-vesicular particles, and detection of mtDNA or mitochondrial proteins does not itself establish intravesicular localization. The Minimal Information for Studies of Extracellular Vesicles 2023 (MISEV2023) recommendations therefore emphasize rigorous assessment of EV purity, co-isolates, and cargo localization [149]. Future studies will require organ-specific EV/cargo tracing, recipient-cell mapping, and causal perturbation of EV biogenesis or uptake. Representative signals are summarized in Table 1. 

### 5.5. Evidence Synthesis: Direct Signaling, Shared Drivers, and Hemodynamic Feedback

Current evidence does not support a single uniform mitochondrial stress loop linking MASLD and HF. Rather, three non-exclusive mechanisms are likely to coexist. First, obesity, insulin resistance, type 2 diabetes, hypertension, and systemic inflammation can impose parallel metabolic and inflammatory stress on the liver and heart, particularly in metabolically burdened HFpEF phenotypes [6,150].

Second, selected forms of liver-to-heart communication are supported to varying degrees, although their mechanisms and evidence levels differ. Hepatic ketogenesis provides a physiological substrate pathway to the myocardium, source-resolved experimental studies support FGF21-mediated hepato-cardiac endocrine signaling in selected cardiac injury models, and hepatocyte-derived EVs can affect extrahepatic vascular and immune cells. The latter, however, does not by itself establish EV-mediated liver-to-heart signaling. A complete causal pathway linking MASLD-associated hepatic mitochondrial injury to the release of a defined mitochondria-associated mediator, engagement of a specific cardiac recipient cell or receptor, development of a defined HF phenotype, and reversal by mediator or pathway blockade has not yet been demonstrated for most candidate signals.

Third, the most firmly established HF-to-liver pathway remains predominantly hemodynamic. Reduced cardiac output, hepatic hypoperfusion, elevated right-sided filling pressures, venous congestion, and systemic inflammatory or neurohumoral activation can induce hepatic hypoxia and secondary mitochondrial injury. These mechanisms are particularly relevant to advanced HFrEF, right-sided HF, and acute decompensation, but congestive hepatopathy or hypoxic liver injury should not be equated with histological progression of MASLD/MASH.

**Figure 3:**
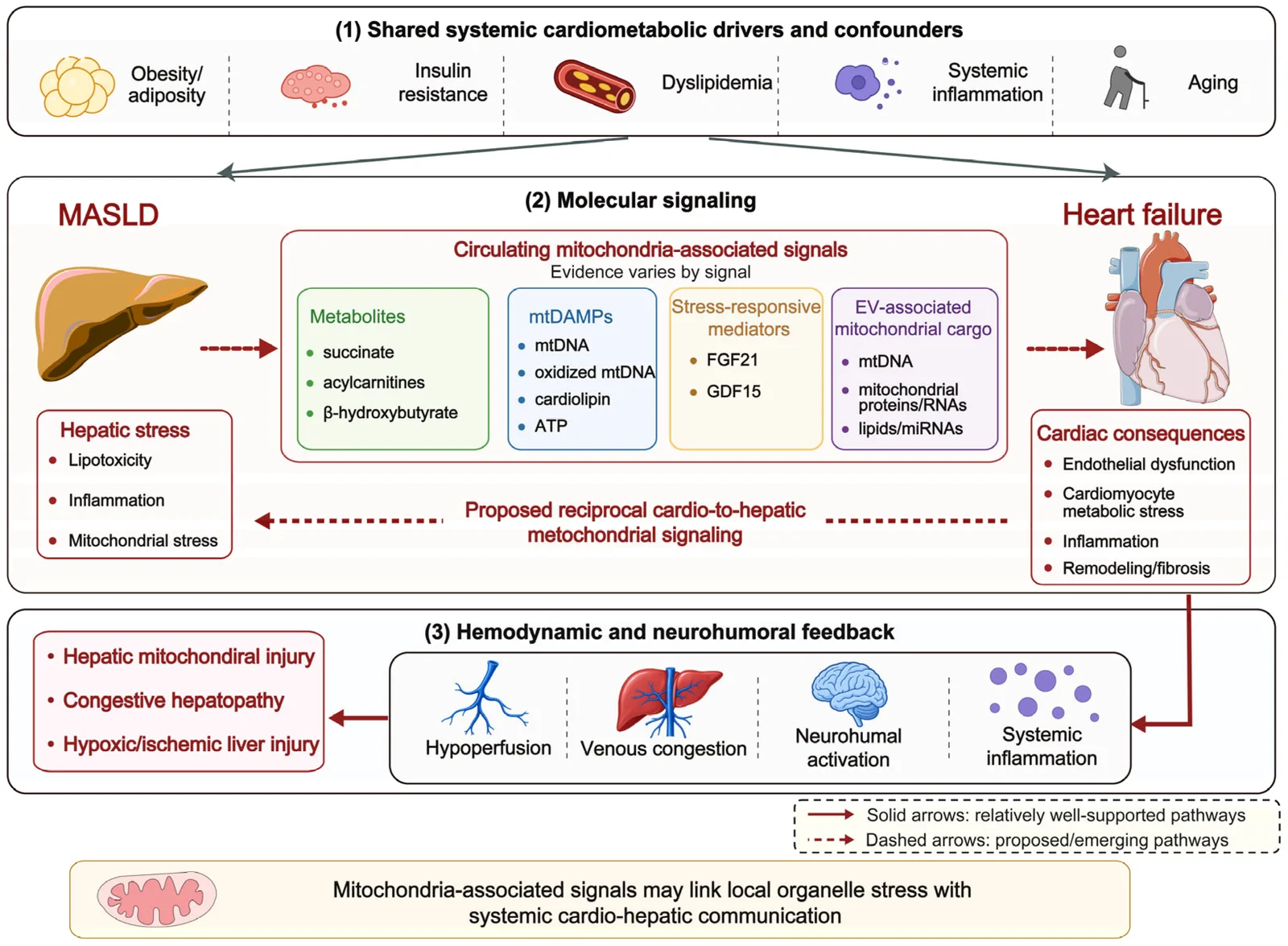
Established and proposed cardio-hepatic pathways involving mitochondria-associated signals. Shared cardiometabolic drivers contribute to mitochondrial stress in both MASLD and HF, while metabolites, mtDAMPs, mitokines, and EV-associated cargo may participate in molecular communication. HF can further aggravate hepatic mitochondrial injury through hemodynamic and neurohumoral feedback. Abbreviations: ATP, adenosine triphosphate; EV, extracellular vesicle; FGF21, fibroblast growth factor 21; GDF15, growth differentiation factor 15; HF, heart failure; MASLD, metabolic dysfunction-associated steatotic liver disease; miRNA, microRNA; mtDAMPs, mitochondrial damage-associated molecular patterns; mtDNA, mitochondrial DNA; RNA, ribonucleic acid.

Current evidence therefore supports a layered network of shared systemic drivers, organ-intrinsic mitochondrial stress, selected source-resolved endocrine communication, candidate extracellular transfer of mitochondrial cargo, and phenotype-specific hemodynamic feedback rather than a single established bidirectional mitochondrial signaling pathway between MASLD and HF (Figure 3).

## 6. Clinical Interpretation of Mitochondria-Associated Biomarkers

Circulating mitochondria-associated molecules may serve as indicators of metabolic or cellular stress, but their biomarker value should be distinguished from their mechanistic roles as signaling mediators. mtDNA, acylcarnitines, ketone bodies, and EV-associated mitochondrial components differ in tissue specificity, stability, and sensitivity to pre-analytical conditions; therefore, altered circulating levels may reflect tissue injury, adaptive metabolism, impaired clearance, or systemic cardiometabolic stress rather than direct liver–heart communication.

Circulating mtDNA illustrates these limitations. Plasma mtDNA is elevated in steatohepatitis and severe acute HF; in the latter, higher levels have been associated with short-term mortality [27,151]. However, measurements are highly sensitive to sample handling. Platelet activation can release cell-free mitochondria and mtDNA, making residual platelets and ex vivo activation important sources of variability [152]. Anticoagulant choice, processing time, centrifugation, storage, freeze–thaw cycles, and hemolysis should therefore be carefully controlled [153]. Conventional assays also cannot reliably distinguish hepatic, cardiac, vascular, immune-cell, or platelet sources.

Metabolite biomarkers are influenced by both systemic metabolism and clearance. The liver and skeletal muscle contribute differently to the circulating acylcarnitine pool during fasting and exercise [154], while renal function also affects multiple acylcarnitine species [155]. Ketone bodies similarly vary with nutritional status and physical activity [156]. Accordingly, acylcarnitine and β-hydroxybutyrate levels should be interpreted under standardized metabolic conditions and in relation to renal function and disease phenotype. In HF, increased β-hydroxybutyrate may reflect adaptive ketone utilization rather than greater mitochondrial injury [38,117].

EV-associated mitochondrial biomarkers pose additional analytical challenges because EV isolation methods differ in recovery efficiency and may co-isolate lipoproteins, protein aggregates, platelets, or other extracellular particles. Detection of mtDNA or mitochondrial proteins in EV-enriched fractions therefore does not establish intravesicular localization or tissue origin. The MISEV2023 recommendations emphasize standardized sample processing, EV characterization, assessment of co-isolates, cargo localization, and appropriate normalization [149]. More broadly, cross-sectional measurements cannot establish whether a circulating biomarker is a cause or consequence of disease or determine the direction of inter-organ signaling. Future studies should therefore prioritize standardized biospecimen handling, longitudinal sampling, adjustment for renal and metabolic status, and stratification by MASLD stage and HF phenotype. At present, these biomarkers are better suited to complementary phenotyping and longitudinal monitoring than to establishing organ-specific causality.

## 7. Therapeutic Implications

Therapeutic strategies relevant to the MASLD–HF overlap can be considered at four levels: systemic metabolic and hemodynamic unloading, liver-directed reduction of metabolic stress, direct restoration of mitochondrial resilience, and containment of inflammatory stress signals. Importantly, most available evidence derives from separate MASLD/MASH or HF populations rather than from patients with a specifically defined MASLD-associated HF phenotype. Representative strategies and their translational status are summarized in Table 2.

Sodium–glucose cotransporter 2 (SGLT2) inhibitors provide established clinical benefit across the HF ejection-fraction spectrum and may indirectly reduce mitochondrial stress through systemic metabolic and hemodynamic unloading, altered substrate utilization, and reduced oxidative and inflammatory stress [157,158]. Incretin-based therapies provide a complementary strategy by reducing obesity, insulin resistance, adipose lipolysis, and hepatic lipid influx. Semaglutide improves symptoms and physical function in obesity-related HFpEF and has demonstrated histological efficacy in MASH [159,160]. Tirzepatide has shown efficacy in phase 2 MASH and clinical benefit in HFpEF with obesity, although these findings do not establish a direct mitochondrial or liver-to-heart mechanism [161,162].

Liver-directed metabolic therapies may further reduce hepatic metabolic stress without necessarily constituting direct cardio-hepatic mitochondrial interventions. Resmetirom, a liver-directed thyroid hormone receptor-β agonist, improves MASH resolution and liver fibrosis outcomes in the phase 3 MAESTRO-NASH trial [163]. FGF21 analogues represent another metabolic therapeutic approach. Pegozafermin and efruxifermin have shown antifibrotic activity in phase 2b MASH studies, and both have progressed to phase 3 development [164,165]. More recent 96-week phase 2b data support sustained antifibrotic activity of efruxifermin in noncirrhotic MASH, whereas a separate trial in compensated MASH cirrhosis did not meet its 36-week fibrosis endpoint [166,167]. Dedicated HF outcome evidence for FGF21 analogues remains lacking.

Direct mitochondria-centered interventions remain substantially less mature. Strategies targeting mitochondrial redox balance, nicotinamide adenine dinucleotide (NAD^+^) availability, mitochondrial biogenesis, mitophagy, and lysosomal quality control have shown mechanistic promise, but HF clinical evidence remains limited. Elamipretide, a mitochondrial cardiolipin-binding peptide, received accelerated U.S. Food and Drug Administration approval in 2025 for Barth syndrome in patients weighing at least 30 kg; however, a phase 2 study in stable HFrEF showed no significant improvement in left ventricular end-systolic volume, and its efficacy in HF remains unproven [168,169]. Preclinical strategies targeting mtDNA release or sensing through TLR9 and cGAS–STING, SUCNR1 signaling, or EV-mediated cargo transfer remain exploratory. NLRP3 inhibition has reached early clinical evaluation in HFrEF, but available evidence remains limited to early-phase safety and exploratory biological or clinical signals rather than definitive therapeutic efficacy [170]. The relevant dapansutrile study was a short phase 1b trial in stable HFrEF and primarily established safety and tolerability, with exploratory efficacy signals. However, innate immune pathways targeted by these approaches also contribute to antimicrobial host defense and immune surveillance [171,172]. Prolonged systemic blockade may therefore increase susceptibility to infection or impair protective antitumor immunity, highlighting the need to establish long-term safety and tissue-selective targeting before chronic clinical use. These approaches should aim to restore mitochondrial balance rather than indiscriminately suppress ROS or force mitochondrial turnover.

Overall, mitochondrial dysfunction is better viewed as a therapeutic convergence point rather than as a single drug target or an established liver-to-heart pathway. Current cardiometabolic and liver-directed therapies primarily reduce upstream metabolic or hemodynamic stress, whereas direct mitochondria-centered and stress-signal interventions remain largely investigational. Clinical improvement should therefore not be interpreted as evidence of a mitochondrial or liver-to-heart mechanism unless mechanistic target engagement is demonstrated.

**Table 2:** Therapeutic strategies relevant to mitochondrial dysfunction in the MASLD–HF overlap.

Strategy or Target	Representative Agents or Targets	Principal Action Relevant to the Cardio-Hepatic Axis	Evidence Status	Key Limitations
**Clinical-stage systemic or organ-directed strategies**				
SGLT2 inhibition	Dapagliflozin; empagliflozin	Metabolic, cardiorenal, and hemodynamic unloading	Established HF benefit across EF phenotypes [157,158,173]	Mainly indirect mitochondrial effects; no dedicated MASLD–HF trial
Incretin-based therapy	Semaglutide; tirzepatide	Reduces obesity, insulin resistance, hepatic lipid burden	Clinical efficacy in MASH and obesity-related HFpEF [159,160,161,162]	Phenotype-dependent benefit; direct mitochondrial mechanism unproven
THR-β activation	Resmetirom	Improves hepatic lipid metabolism and reduces MASH activity	Phase 3 efficacy in MASH with fibrosis [163]	Liver-directed; HF outcome evidence lacking
FGF21 modulation	Pegozafermin; efruxifermin	Improves hepatic and systemic metabolic homeostasis	Phase 2 efficacy in MASH [164,165,166,167]	HF outcome evidence lacking; effects may vary by MASH stage
**Mitochondria- and stress-targeted strategies with limited clinical evidence**				
Cardiolipin modulation	Elamipretide	Supports mitochondrial membrane integrity and energetics	Approved for Barth syndrome; neutral phase 2 HFrEF trial [168,169]	MASLD–HF efficacy unproven
NAD^+^ restoration	Nicotinamide riboside	Supports mitochondrial redox and energetics	Early clinical target engagement in HFrEF [174]	Efficacy, dosing, and tissue specificity unresolved
Mitochondrial quality control	AMPK; PINK1/Parkin pathway	Promotes mitochondrial turnover and quality control	Predominantly preclinical cardiac evidence [175]	Context-dependent; requires intact lysosomal flux
Inflammatory stress-signal blockade	NLRP3 inhibition	Limits inflammatory amplification	Phase 1b HFrEF safety/pharmacodynamic evidence [170]	Clinical efficacy, long-term safety, and tissue selectivity unproven

Representative systemic, organ-directed, and mitochondrial/stress-targeted strategies are summarized according to their cardio-hepatic actions, evidence status, and major limitations. Evidence ranges from established clinical benefit to early clinical or preclinical findings, and does not imply efficacy specifically in MASLD-associated HF. No strategy has yet been specifically validated for MASLD-associated HF as a unified clinical entity. Abbreviations: AMPK, AMP-activated protein kinase; EF, ejection fraction; FGF21, fibroblast growth factor 21; HF, heart failure; HFpEF, heart failure with preserved ejection fraction; HFrEF, heart failure with reduced ejection fraction; MASH, metabolic dysfunction-associated steatohepatitis; MASLD, metabolic dysfunction-associated steatotic liver disease; NAD^+^, nicotinamide adenine dinucleotide; NLRP3, NLR family pyrin domain-containing 3; PINK1, PTEN-induced kinase 1; SGLT2, sodium–glucose cotransporter 2; THR-β, thyroid hormone receptor β.

## 8. Clinical Challenges and Future Directions

### 8.1. Disease-Stage and Phenotype Heterogeneity

Both MASLD and HF are clinically and biologically heterogeneous, and this heterogeneity remains a major challenge in translating mitochondria-related mechanisms into a coherent cardio-hepatic framework. MASLD spans steatosis, MASH, progressive fibrosis, and cirrhosis, whereas HF encompasses metabolically and hemodynamically distinct phenotypes, including obesity-related HFpEF, HFrEF, right-sided HF, and acute decompensated HF. Clinical data indicate that MASLD is associated with an increased risk of incident HF, particularly HFpEF [6,176], but such associations do not imply a uniform mitochondrial mechanism across disease stages or HF phenotypes. Importantly, metabolic liver injury should be distinguished from hepatic injury secondary to cardiac dysfunction. Chronic right-sided HF can cause passive venous congestion and congestive hepatopathy, whereas acute reductions in cardiac output or systemic hypoxemia can result in hypoxic liver injury; neither process should be equated with the histological progression of MASLD/MASH [177,178]. Future studies should therefore stratify patients according to both MASLD stage and HF phenotype and avoid extrapolating mitochondrial phenotypes observed in a single disease stage or experimental model to the entire MASLD–HF spectrum.

### 8.2. Establishing Source-to-Recipient Causality

A more fundamental challenge is to establish whether a candidate mitochondria-associated signal truly mediates inter-organ communication. Intracellular activation of mtDNA-dependent inflammatory pathways within hepatocytes or cardiac cells establishes organ-intrinsic stress signaling but does not by itself demonstrate direct inter-organ communication between the liver and heart. Future mechanistic studies should define the source cell, release mechanism, and circulating form of the candidate mediator; identify the recipient cell and its receptor or uptake pathway; and determine whether source-specific manipulation or blockade of the mediator alters the distant-organ phenotype. Source-resolved studies provide useful methodological precedents. For example, tissue-specific genetic models have provided evidence that liver-derived FGF21 contributes to cardiac signaling during pressure overload [91]. In another inter-organ system, EVs released from metabolically stressed adipocytes were shown to carry mitochondrial material into the circulation and deliver it to cardiomyocytes [28]. The latter provides proof of principle for source-to-recipient mitochondrial cargo transfer rather than direct evidence of liver-to-heart signaling. Accordingly, future studies should combine tissue- or cell-specific perturbation with source tracing, recipient-cell mapping, and mediator or receptor blockade to distinguish cell-autonomous and local paracrine mechanisms from true inter-organ signaling.

### 8.3. Clinical Validation and Biomarker Standardization

Clinical translation will also require a shift from predominantly cross-sectional associations toward standardized, longitudinal, and phenotype-resolved study designs. Circulating mtDNA illustrates this challenge: elevated concentrations have been associated with mortality in severe acute HF [151], but circulating mtDNA measurements can be substantially affected by platelet activation and the release of cell-free mitochondria [152]. Metabolomic measurements are likewise sensitive to pre-analytical blood-processing conditions [153]. Circulating acylcarnitines are influenced by tissue source, fasting, and exercise [154], while renal function can alter the concentrations of multiple circulating metabolites [155]. EV-associated biomarkers introduce additional methodological challenges related to particle isolation, co-isolates, cargo localization, and normalization; these issues are addressed in the MISEV2023 recommendations [149]. Future clinical studies should therefore incorporate standardized biospecimen handling, repeated sampling, paired cardiac and hepatic phenotyping, and longitudinal clinical outcomes. Such designs may help distinguish nonspecific disease markers from adaptive responses and candidate pathogenic mediators, while reducing reliance on single time-point measurements to infer directionality.

Overall, progress in this field will depend less on expanding the list of putative mitochondrial distress signals than on strengthening the evidence for existing candidates. Priority should be given to pathways with clearly defined cellular sources and recipients, defined circulating forms and biological activity at physiologically relevant concentrations or exposure levels, and causal support from source-specific perturbation or pathway blockade. Integrating such mechanistic evidence with longitudinal, stage- and phenotype-resolved clinical studies will be essential to determine which mitochondria-associated signals have mechanistic and clinical relevance to cardio-hepatic communication.

## 9. Conclusions

MASLD and HF frequently coexist within a shared cardiometabolic setting [1,2,6]. However, current evidence supports stage- and phenotype-dependent mitochondrial remodeling rather than a single mitochondrial phenotype common to both diseases. In the liver, increased oxidative metabolism may initially compensate for excess substrate supply, whereas this mitochondrial flexibility becomes impaired with progression toward steatohepatitis [23,24]. In the failing myocardium, reduced energetic reserve and altered substrate utilization, including enhanced ketone oxidation in advanced HF, reflect a distinct pattern of energetic stress and metabolic remodeling [36,38]. These findings support convergent but organ-specific mitochondrial maladaptation and should not, by themselves, be interpreted as evidence of direct heart–liver signaling.

Mitochondrial dysfunction can nevertheless alter the extracellular availability or biological activity of metabolites, mtDAMPs, stress-responsive mediators, and EV-associated cargo. The strength of evidence differs substantially among these signal classes. Hepatocyte-derived mtDNA-containing microparticles can activate TLR9-dependent inflammatory signaling in steatohepatitis [27], whereas source-resolved evidence supports liver-derived FGF21 signaling to the heart during pressure overload [91]. By contrast, EV-mediated mitochondrial cargo transfer has been demonstrated in other inter-organ systems but does not yet establish a causal liver-to-heart pathway in MASLD-associated HF [28]. HF-related venous congestion and hypoperfusion can also independently contribute to hepatic injury [177,178]. These hemodynamic mechanisms should be distinguished from direct mitochondria-associated inter-organ signaling.

From a translational perspective, therapies with established clinical benefit currently act predominantly by reducing upstream metabolic or hemodynamic stress or by targeting hepatic metabolic disease, whereas direct mitochondria-centered interventions and mitochondrial stress-signal blockade remain substantially less mature. Accordingly, mitochondrial dysfunction is best viewed as a therapeutic and mechanistic convergence point, rather than as a single drug target or a proven liver-to-heart pathway. Further progress will require integration of MASLD stage and HF phenotype with source- and recipient-cell resolution, standardized longitudinal biomarker assessment, and causal validation of candidate mediators. Such an approach should help determine which mitochondria-associated signals are mechanistically informative and clinically actionable in the MASLD–HF overlap.

## Figures and Tables

**Table 1:** Representative mitochondria-associated signals relevant to cardio-hepatic communication.

Signal	Source Attribution	Principal Mechanism	Functional Interpretation	Evidence Status
Succinate	Multisource	SUCNR1 signaling	Context-dependent metabolic signaling	Mechanistic; source unresolved [108,109,110]
Acylcarnitines	Multisource	Signaling pathway not established	Metabolic readout of incomplete FAO	Associative; source unresolved [111,112,113,114,115]
β-Hydroxybutyrate	Predominantly hepatic	Ketone oxidation; NLRP3 inhibition	Adaptive substrate and immunometabolic signal	Physiological; signaling context-dependent [38,116,117,118]
mtDNA/ox-mtDNA	Hepatic/hepatocyte-derived in selected models *	TLR9; cytosolic cGAS–STING; NLRP3	Sterile inflammatory signaling	Source-resolved; cardiac transfer unproven [27,120,121,122,123,124,125,126,127,128]
FGF21	Predominantly hepatic	β-Klotho/FGFR signaling	Stress-responsive endocrine signaling	Inter-organ; cardiac causality context-dependent [91,133,134,135,136,137]
GDF15	Multisource	GFRAL–RET signaling	Systemic neuroendocrine stress signaling	Systemic endocrine; cardiac signaling unproven [138,139,140,141,142]
EV-associated mitochondrial cargo	Hepatic/hepatocyte-derived in selected models *	Cellular uptake; TLR9/cytosolic nucleic-acid sensing	Transport of mitochondrial material	Source-resolved; cardiac effects unproven [27,143,144,145,146,147,148,149]

Signals are summarized by source, mechanism, functional interpretation, and evidence status. Evidence status does not necessarily imply direct liver-to-heart causality. * Source attribution is based on selected models and does not indicate an exclusive tissue source. Circulating levels are not organ-specific and may be influenced by systemic and clinical factors. Abbreviations: cGAS, cyclic GMP–AMP synthase; EV, extracellular vesicle; FAO, fatty acid oxidation; FGF21, fibroblast growth factor 21; FGFR, fibroblast growth factor receptor; GDF15, growth differentiation factor 15; GFRAL, GDNF family receptor alpha-like; mtDNA, mitochondrial DNA; NLRP3, NLR family pyrin domain-containing 3; ox-mtDNA, oxidized mitochondrial DNA; RET, rearranged during transfection; STING, stimulator of interferon genes; SUCNR1, succinate receptor 1; TLR9, Toll-like receptor 9.

## Data Availability

No new data were created or analyzed in this study. Data sharing is not applicable to this article.

## Abbreviations

The following abbreviations are used in this manuscript:

AMPK	AMP-activated protein kinase
ATF4	activating transcription factor 4
ATF5	activating transcription factor 5
ATP	adenosine triphosphate
BAK1	BCL-2 antagonist/killer 1
BAX	BCL-2-associated X protein
BNIP3	BCL2/adenovirus E1B 19 kDa protein-interacting protein 3
BNIP3L	BCL2/adenovirus E1B 19 kDa protein-interacting protein 3-like
cGAS	cyclic GMP-AMP synthase
CHOP	C/EBP homologous protein
CVD	cardiovascular disease
DAMP	damage-associated molecular pattern
DCM	dilated cardiomyopathy
DELE1	DAP3-binding cell death enhancer 1
DRP1	dynamin-related protein 1
EF	ejection fraction
eIF2α	eukaryotic translation initiation factor 2 alpha
ER	endoplasmic reticulum
ETC	electron transport chain
EV	extracellular vesicle
FAO	fatty acid oxidation
FGF21	fibroblast growth factor 21
FGFR	fibroblast growth factor receptor
FPR1	formyl peptide receptor 1
FUNDC1	FUN14 domain-containing protein 1
GDF15	growth differentiation factor 15
GFRAL	GDNF family receptor alpha-like
GPR91	G protein-coupled receptor 91
HF	heart failure
HFpEF	heart failure with preserved ejection fraction
HFrEF	heart failure with reduced ejection fraction
HRI	heme-regulated eIF2α kinase
IL-6	interleukin-6
ISR	integrated stress response
LC3	microtubule-associated protein 1 light chain 3
MASH	metabolic dysfunction-associated steatohepatitis
MASLD	metabolic dysfunction-associated steatotic liver disease
MFF	mitochondrial fission factor
MFN1	mitofusin 1
MFN2	mitofusin 2
MISEV2023	Minimal Information for Studies of Extracellular Vesicles 2023
miRNA	microRNA
mitoISR	mitochondrial integrated stress response
MIZ1	MYC-interacting zinc-finger protein 1
mtDAMP	mitochondrial damage-associated molecular pattern
mtDNA	mitochondrial DNA
mTORC1	mechanistic target of rapamycin complex 1
NAD^+^	oxidized nicotinamide adenine dinucleotide
NADPH	reduced nicotinamide adenine dinucleotide phosphate
NAFLD	nonalcoholic fatty liver disease
NASH	nonalcoholic steatohepatitis
NDP52	nuclear dot protein 52
NF-κB	nuclear factor-κB
NIX	common name for BCL2-interacting protein 3-like (BNIP3L)
NLRP3	NLR family pyrin domain-containing 3
NOX4	nicotinamide adenine dinucleotide phosphate oxidase 4
NRF2	nuclear factor erythroid 2-related factor 2
OMA1	OMA1 zinc metallopeptidase
OPA1	optic atrophy 1
OPTN	optineurin
OXPHOS	oxidative phosphorylation
p66Shc	66-kDa SHC-transforming protein 1 isoform
PCr/ATP	phosphocreatine-to-ATP ratio
PDH	pyruvate dehydrogenase
PGC-1α	peroxisome proliferator-activated receptor-γ coactivator 1α
PINK1	PTEN-induced kinase 1
PPARγ	peroxisome proliferator-activated receptor-γ
RET	rearranged during transfection
RNA	ribonucleic acid
ROS	reactive oxygen species
SGLT2	sodium–glucose cotransporter 2
SOD2	superoxide dismutase 2
SQSTM1/p62	sequestosome 1/p62
STAT3	signal transducer and activator of transcription 3
STING	stimulator of interferon genes
SUCNR1	succinate receptor 1
TCA	tricarboxylic acid
TFAM	mitochondrial transcription factor A
THR-β	thyroid hormone receptor β
TLR9	toll-like receptor 9
TNF-α	tumor necrosis factor-α
UPRmt	mitochondrial unfolded protein response
VDAC	voltage-dependent anion channel
ΔΨm	mitochondrial membrane potential
